# *De novo* assembly and comparative analysis of the first complete mitogenome in *Distylium* (*Distylium racemosum*)

**DOI:** 10.3389/fpls.2025.1586341

**Published:** 2025-05-15

**Authors:** Yaling Wang, Zhongxiao Zhang, Xinru Chen, Honghe Li, Chi Ma, Penghui Guo

**Affiliations:** School of Life Sciences and Engineering, Northwest Minzu University, Lanzhou, China

**Keywords:** *Distylium racemosum*, mitochondrial genome, phylogenetic analysis, RNA editing events, comparative analysis

## Abstract

The genus *Distylium* (Hamamelidaceae) is highly valued for its applications in ornamental horticulture, industry, and construction. Although plastid genomes (plastomes) of multiple *Distylium* species have been characterized, no mitochondrial genomes (mitogenomes) have been reported for this genus. In this study, we assembled and annotated the complete mitogenome of *Distylium racemosum* using HiFi sequencing data. The mitogenome comprises a longer circular chromosome and a shorter linear chromosome (904,264 bp in total length), revealing a structurally complex conformation. We annotated 67 genes, including 43 protein-coding genes (PCGs), 21 tRNA genes, and three rRNA genes. Analyses identified exceptionally high repetitive sequence content, with 304 simple sequence repeats, 1,508 dispersed repeats, and 50 tandem repeats, representing the highest repeat content among Saxifragales mitogenomes to date. Additionally, 49 mitochondrial plastid DNA sequences were detected, including only one complete plastid-derived gene (*trnC-GCA*) transferred to the mitogenome. We predicted 697 RNA editing sites across 42 PCGs, further underscoring the genome’s dynamic evolution. Phylogenetic reconstruction based on mitogenomes and plastomes from 18 species indicated *D. racemosum* occupied a basal position within Saxifragales, which is consistent with the APG IV classification system. This study provides the first comprehensive mitogenomic resource for the *Distylium* genus, offering valuable insights for molecular classification, species identification, and germplasm conservation of *Distylium* plants.

## 1 Introduction

*Distylium racemosum*, a flowering plant in the genus *Distylium* (Hamamelidaceae), is endemic to Asia. This evergreen shrub or small tree inhabits warm-temperate lowland forests, distinguished by dense branching, persistent dark-green foliage, and a compact growth form (Dong et al., 2021). Its ornamental appeal is enhanced by clusters of small red flowers produced in spring. The *Distylium* species exhibits notable resistance to airborne pollutants, including sulfur dioxide and chlorine, making it particularly suitable for urban greening in industrial and mining zones. Notably, *Distylium chinense* demonstrates exceptional ecological adaptability, serving as a key species for soil conservation and embankment stabilization (Liu et al., 2014; Xiang et al., 2020). *Distylium* species also engages in a unique ecological interaction with the aphid *Nipponaphis distychii*, which induces large gall formation on leaves. These galls accumulate tannins, compounds historically utilized as natural dyes (Ismayati et al., 2024). Furthermore, the wood of *Distylium* is exceptionally dense and durable, rendering it valuable for construction applications such as housing and vehicle manufacturing (Yagi et al., 2019).

Mitochondria, semi-autonomous organelles possessing independent genetic material and distinct replication and expression systems, are critical for cellular respiration and metabolic regulation (Dyall et al., 2004; Gray et al., 1999; van Loo et al., 2002). Unlike animal mitochondrial genomes (mitogenomes, typically 10–20 kb) and plant plastid genomes (plastomes, generally 100–200 kb), which exhibit size conservation (Liu et al., 2022; Wu et al., 2022), plant mitogenomes display substantial size variation, ranging from 66 kb in *Viscum scurruloideum* to 18.99 Mb in *Cathaya argyrophylla* (Huang et al., 2024; Skippington et al., 2015). These genomes are pivotal for elucidating cytoplasmic male sterility mechanisms and developing molecular breeding applications (Han et al., 2024; Xiao et al., 2020). However, plant mitogenome assembly remains technically challenging due to extreme structural plasticity, long repetitive sequences, and frequent horizontal transfer of nuclear mitochondrial DNA (NUMTs) and mitochondrial plastid DNA (MTPTs) (Han et al., 2022; Sun et al., 2024). To date, over 34,000 complete plastomes have been reported in Chloroplast Genome Information Resource (CGIR) (https://ngdc.cncb.ac.cn/cgir), while the NCBI Nucleotide database (https://www.ncbi.nlm.nih.gov/) currently contains fewer than one thousand complete mitogenomes for plants (last accessed: February 28th, 2025) (Huang et al., 2025; Wang J. et al., 2024). This disparity highlights the complexity and technical challenges associated with plant mitogenome assembly and annotation.

Previous plastome sequencing of 12 *Distylium* species has established a phylogenomic framework for understanding the genus’s evolution (Dong et al., 2021). Despite these advances, comprehensive genomic resources for *Distylium* (particularly nuclear and mitochondrial genomes) remain critically limited. To date, the mitogenomes of *Distylium* species remain entirely unexplored, and their phylogenetic relationships based on mitogenomic data are still uncleared. While mitogenomes have been assembled for numerous Saxifragales species, only two unannotated mitogenomes from Hamamelidaceae (*Rhodoleia championii* and *Loropetalum chinense*) are available in the NCBI database, with none reported for *Distylium*. This gap underscores the necessity to prioritize mitogenome sequencing and annotation in Hamamelidaceae. Such initiatives will advance our understanding of evolutionary trajectories within the family and improve phylogenomic resolution across Saxifragales.

In this study, we successfully assembled and annotated the mitogenome of *D. racemosum*, establishing the inaugural mitochondrial genomic resource for the *Distylium* genus. We comprehensively characterized the mitogenome’s structural features, repeat content, RNA editing sites and MTPTs, filling a critical gap in genomic data for this genus. Comparative genomic analyses and phylogenies reconstructed from mitochondrial and plastid protein-coding genes (PCGs) further clarified evolutionary relationships. These results provide foundational resources for molecular taxonomy, species discrimination, and germplasm conservation in *Distylium*, while elucidating broader evolutionary dynamics within Saxifragales.

## 2 Materials and methods

### 2.1 DNA extraction and sequencing

Fresh leaves of *D. racemosum* were collected from Nanjing Forestry University (Nanjing, China; 32°08′ N, 118°82′ E) and immediately frozen at −80°C for downstream analysis. High-quality genomic DNA was extracted using the CTAB method (Arseneau et al., 2017). DNA integrity and purity were verified via 1.0% agarose gel electrophoresis and quantified using a NanoDrop 2000 spectrophotometer (Thermo Fisher Scientific) (Shi et al., 2023). Sequencing libraries were prepared with the SMRTbell Express Template Prep Kit 2.0 (PacBio Biosciences, California, USA) using high-molecular-weight DNA. High-fidelity (HiFi) sequencing data was subsequently performed on the PacBio Revio platform.

### 2.2 Assembly and annotation of *D. racemosum* mitogenome

The HiFi sequencing data were processed with PMAT v2.0 (Bi et al., 2024a) to assemble the *D. racemosum* mitogenome using parameters ‘-t hifi -m -F 0.2 -T 50’ in ‘autoMito’ mode. The raw assembly graph was resolved and visualized with Bandage (Wick et al., 2015). Annotation of the *D. racemosum* mitogenome was carried out using the online program PMGA (http://47.96.249.172:16084/home) (Li et al., 2024). Then the rRNA genes (rRNAs) and tRNA genes (tRNAs) were verified using BLASTn and tRNAscan-SE v2.0, respectively (Camacho et al., 2009; Chan et al., 2021; Chen et al., 2015). Finally, the genome map of *D. racemosum* mitogenome was visualized using PMGmap with default parameters (Zhang et al., 2024).

### 2.3 Detection of repeat sequences and prediction of RNA editing sites

Simple sequence repeats (SSRs) in the *D. racemosum* mitogenome were identified using the online platform MISA (https://webblast.ipk-gatersleben.de/misa/) (Beier et al., 2017). The thresholds for the minimum number of repetitions were set as follows: 10 for mononucleotides, 5 for dinucleotides, 4 for trinucleotides, and 3 for tetranucleotides, pentanucleotides, and hexanucleotides. Dispersed repeats were detected using the program vmatch-2.3.1with parameters ‘-d -p -h 3 -l 30 -best 5000 -noscore -noidentity -absolute’ (Kurtz et al., 2001). Additionally, tandem repeats in the mitogenome were identified using Tandem Repeats Finder v4.09 (https://tandem.bu.edu/trf/trf.html) with default parameters (Benson, 1999). All repeat elements were manually verified and then visualized using the ggplot2 package in R.

RNA editing, a post-transcriptional process involving the insertion, deletion, or substitution of nucleotides in RNA transcripts, plays a critical role in expanding transcriptional and functional diversity within mitochondrial genes (Fan et al., 2019). To construct reference sequences, we extracted coding sequences for all PCGs from the *D. racemosum* mitogenome (Yang et al., 2023a). Subsequently, the RNA editing sites in the *D. racemosum* mitogenome were predicted using the Deepred-Mt software (Edera et al., 2021), with a probability threshold set at 0.9 to ensure high-confidence identification.

### 2.4 Analysis of MTPTs and collinearity

To identify the MTPTs in *D. racemosum*, the complete plastome of *D. racemosum* was obtained from NCBI with accession number of NC_059886. Subsequently, BLASTn was utilized to detect homologous fragments between the mitogenome and plastome of *D. racemosum* with the following parameters: ‘-word_size 9 -evalue 1e-5 -reward 2 -gapopen 5 -gapextend 2 -penalty -3 -outfmt 6’ (Camacho et al., 2009; Chen et al., 2015). Fragments with a matching rate ≥80% and lengths ≥30 bp were selected for further analysis. The selected fragments were manually annotated to determine their locations within the mitogenome and plastome. The results of MTPTs were finally visualized using Circos (v0.69-5) (Zhang et al., 2013).

To investigate the mitogenome structure of *D. racemosum*, we downloaded three additional Saxifragales mitogenomes (*Paeonia lactiflora*, *Ribes meyeri*, and *Rhodiola tangutica*) from the NCBI database (**Supplementary Table S1**). Collinear blocks were identified using BLASTn with stringent criteria applied: only alignments with a minimum length of ≥200 bp and a minimum identity of ≥80% were retained for further analysis. The results were visualized using NGenomeSyn to facilitate structural comparisons (He et al., 2023).

### 2.5 Phylogenetic analysis

For the construction of phylogenetic trees for both mitochondria and plastids, we obtained the mitogenomes and plastomes of 17 additional species via the NCBI Nucleotide database, with *Sorghum bicolor* designated as the outgroup. Detailed species information and corresponding accession numbers are provided in **Supplementary Table S1**. We extracted 18 shared conserved PCGs from mitogenomes and 59 common conserved PCGs from plastomes using a self-written python script. Then we performed multiple sequence alignment utilizing MAFFT v7.4 (Katoh and Standley, 2013). After trimming the results using trimAl v1.4 (Capella-Gutiérrez et al., 2009), the maximum likelihood (ML) trees were reconstructed using IQ-TREE v2.0.3 with 1000 bootstrap replicates (Minh et al., 2020). The evolutionary model ‘GTR+F+I+G4’ was selected as the fittest model for both mitochondrial and plastid ML trees based on Bayesian Information Criterion (BIC) scores. Finally, the ML tree results were visualized using the online tool iTOL v5 (https://itol.embl.de/) (Letunic and Bork, 2021).

## 3 Results

### 3.1 Characteristics of the *D. racemosum* mitogenome

**Figure 1:**
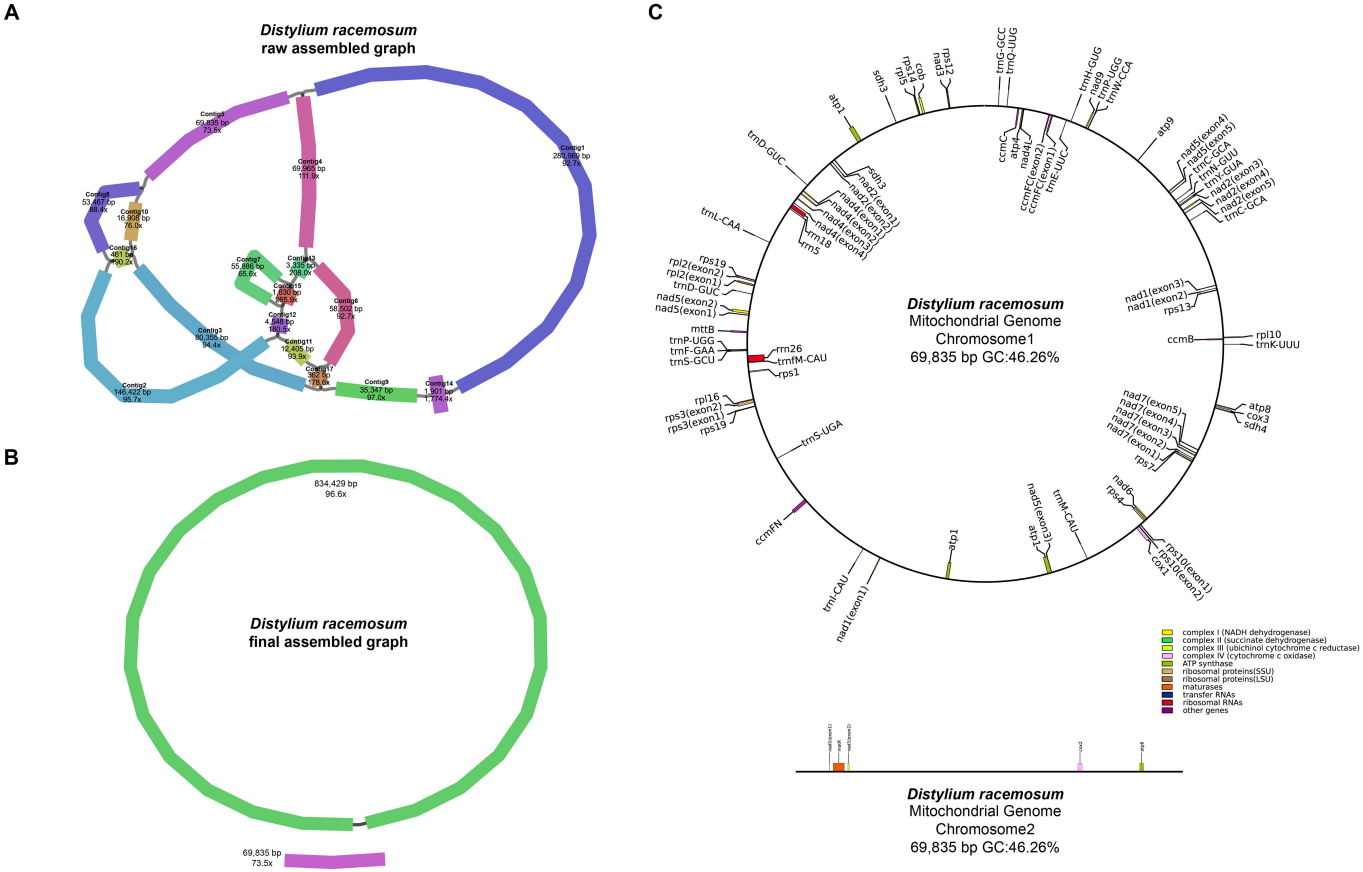
Assembly graphs and genome map of the *Distylium racemosum* mitogenome. **(A)** Raw assembly graph. **(B)** Disentangled assembly graph. **(C)** The genome map of the *Distylium racemosum* mitogenome. Different types of genes were represented by distinct color blocks.

Using the Revio sequencing platform, we generated 865,212 HiFi reads totaling 15.49 Gb, with an N50 value of 14,470 bp (**Table 1**). The mitogenome structure of *D. racemosum* exhibited significant complexity upon disentangling the mitogenome graph using Bandage (**Figures 1A, B**), consisting of a dominant circular molecule (834,429 bp) and a smaller linear fragment (69,835 bp) (**Figure 1C**). The complete mitogenome spans 904,264 bp with 96.9× average coverage, a GC content of 46.28%, and contains 67 genes occupying 5.01% (45,287 bp) of the sequence (**Supplementary Table S2**). These include 43 PCGs (37,845 bp, 4.19%), three rRNA genes (5,861 bp, 0.65%), and 21 tRNA genes (1,581 bp, 0.17%). All 24 core mitochondrial PCGs (*atp1*, *atp4*, *atp6*, *atp8*, *atp9*, *ccmB*, *ccmC*, *ccmFC*, *ccmFN*, *cob*, *cox1*, *cox2*, *cox3*, *matR*, *mttB*, *nad1*, *nad2*, *nad3*, *nad4*, *nad4L*, *nad5*, *nad6*, *nad7* and *nad9*) and 15 variable PCGs (*rpl2*, *rpl5*, *rpl10*, *rpl16*, *rps1*, *rps3*, *rps4*, *rps7*, *rps10*, *rps12*, *rps13*, *rps14*, *rps19*, *sdh3*, and *sdh4*) were identified (**Table 2**). Notable features include three copies of *atp1*, two copies of *rps19* and *sdh3*, and *nad1* presenting on both the circular and linear molecules.

**Table 1:** The length and depth of each assembled contig in *Distylium racemosum*.

Chromosome	Contig name	Coverage (x)	Length (bp)
Mitochondrial chromosome 1	Contig1	92.7	280,969
	Contig2	95.7	146,422
	Contig3	94.4	80,355
	Contig4	111.9	69,965
	Contig6	92.7	58,502
	Contig7	65.6	55,886
	Contig8	88.4	53,467
	Contig9	97.0	35,347
	Contig10	76.0	16,908
	Contig11	93.9	12,405
	Contig12	180.5	4,548
	Contig13	208.0	3,335
	Contig14^a^	1,774.4	1,901
	Contig15	265.9	1,630
	Contig16	190.2	461
	Contig17	178.6	360
Mitochondrial chromosome 2	Contig5	73.5	69,835
Total/Average	17	99.6	904,264

a cp-derived.

**Table 2:** Gene composition in the mitogenome of *Distylium racemosum*.

Group of genes	Name of genes
ATP synthase	*atp1* (×3)*, atp4, atp6, atp8, atp9*
Cytochrome c biogenesis	*ccmB, ccmC, ccmFC, ccmFN*
Ubiquinol cytochrome c reductase	*cob*
Cytochrome c oxidase	*cox1, cox2, cox3*
Maturases	*matR*
Transport membrane protein	*mttB*
NADH dehydrogenase	*nad1* **##, nad2***#, nad3, nad4***, nad4L, nad5**##, nad6, nad7****, nad9
Large subunit of ribosome	rpl2*, rpl5, rpl10, rpl16
Small subunit of ribosome	rps1, rps3*, rps4, rps7, rps10*, rps12, rps13, rps14, rps19 (×2)
Succinate dehydrogenase	*sdh3* (×2)*, sdh4*
Ribosome RNA	*rrn5, rrn18, rrn26*
Transfer RNA	*trnC-GCA* (×2)*, trnD-GUC* (×2)*, trnE-UUC, trnF-GAA, trnfM-CAU, trnG-GCC, trnH-GUG, trnI-CAU, trnK-UUU, trnL-CAA, trnM-CAU*, *trnN-GUU, trnP-UGG* (×2)*, trnQ-UUG*, *trnS-GCU, trnS-UGA, trnW-CCA, trnY-GUA*

“*” labeled the number of *cis*-spliced introns.

“#” labeled the number of *trans*-spliced introns.

“×” labeled the number of genes.

The mitogenome harbors 23 introns (18 *cis*-spliced and 5 *trans*-spliced; **Supplementary Figure S1**), collectively spanning 30,760 bp (3.40% of the genome; **Supplementary Table S2**). A total of 37 PCGs start with ATG, while *cox1*, *nad1*, and *nad4L* use ACG as their start codon. The start codons for *mttB*, *rpl16*, and *rps4* remain unresolved. Stop codons included TAA (21 PCGs), TAG (8), TGA (11), and CGA (3) (**Supplementary Table S3**). The complete mitogenome of *D. racemosum* has been deposited in NCBI Nucleotide database under accessions PQ594873 and PQ594874.

### 3.2 Analysis of repeats

**Figure 2:**
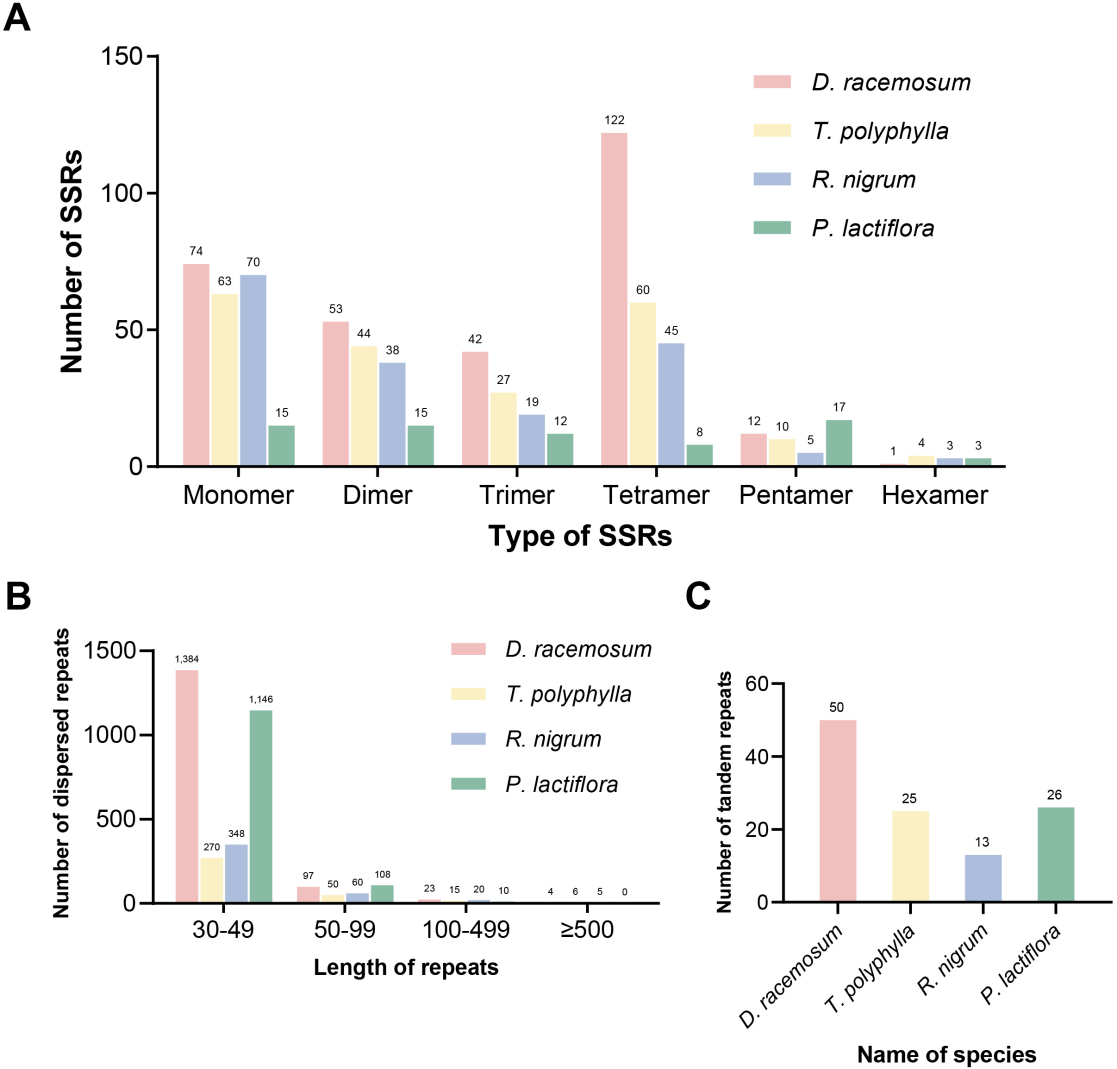
Repeat elements detected in the mitogenomes of four Saxifragales species. **(A)** Type and number of simple sequence repeats in the mitogenomes of four Saxifragales species. **(B)** Type and number of dispersed repeats in the mitogenomes of four Saxifragales species. **(C)** Number of tandem repeats in the mitogenomes of four Saxifragales species.

SSRs were widely distributed throughout the mitochondrial genome. Using the online platform MISA, we identified 304 SSRs, including 74 mononucleotide, 53 dinucleotide, 42 trinucleotide, 122 tetranucleotide, 12 pentanucleotide, and one hexanucleotide repeat units (**Figure 2A**; **Supplementary Figure S2**; **Supplementary Table S4**). Analysis of dispersed repeats revealed 1,508 sequences (≥30 bp) spanning 68,859 bp (7.61% of the total genome length), comprising 748 direct and 760 palindromic repeats (**Supplementary Table S5**). Most of the dispersed repeats (1,481 repeats) were shorter than 100 bp, while four exceeded 500 bp (**Figure 2B**), with the maximum length reaching 6,180 bp (**Supplementary Table S5**). Additionally, 50 tandem repeats (8–50 bp in length) with copy identities ≥75% were detected (**Figure 2C**; **Supplementary Table S6**).

To further characterize repeat element patterns in Saxifragales mitogenomes, we performed comparative analyses of SSRs, dispersed repeats, and tandem repeats across four species: *D. racemosum*, *Tiarella polyphylla*, *Ribes nigrum*, and *P. lactiflora*. The *D. racemosum* mitogenome harbored the highest abundance of all three repeat types. SSR profiling revealed six conserved repeat categories (mono- to hexanucleotide) across all species (**Figure 2A**). The *D. racemosum* mitogenome exhibited the highest number of tetranucleotide repeat units, totaling 122. The number of dispersed repeats varied among species, but the length distributions were conserved: 30–49 bp repeats predominated, while only a few exceeded 500 bp (**Figure 2B**).

### 3.3 Analysis of MTPTs and prediction of RNA editing events

**Figure 3:**
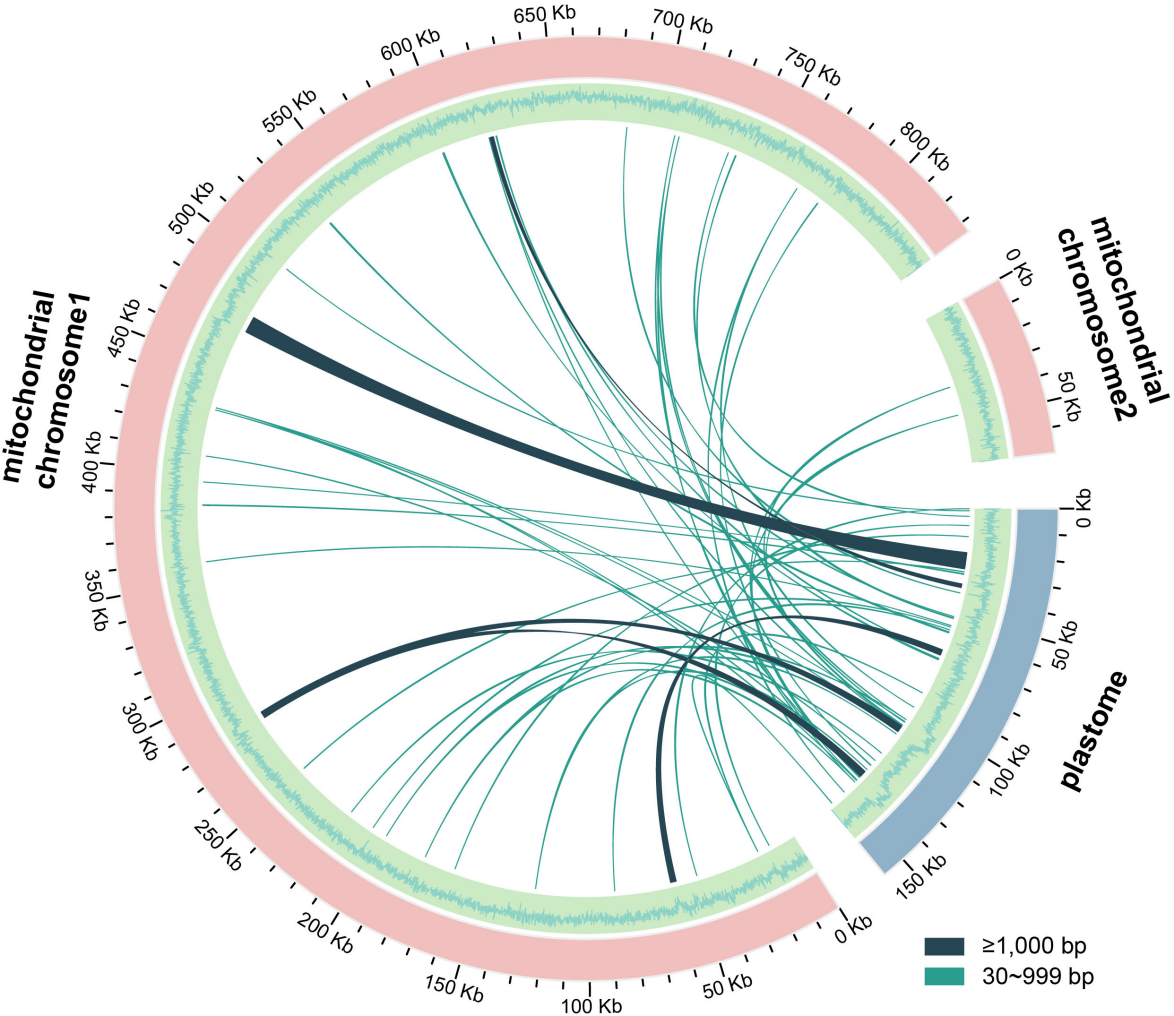
Distribution of mitochondrial plastid DNA sequences (MTPTs) in *Distylium racemosum*. The plastid and mitochondrial genomes of *D. racemosum* were illustrated by pink and blue arcs, respectively. The outer circle depicts the GC content of the two mitochondrial chromosomes and the plastid genome, with adjacent bars indicating the length of MTPTs. Connecting lines between the arcs represented the MTPTs. Detailed information of MTPTs was shown in **Supplementary Table S7**.

A total of 49 MTPTs were identified in the *D. racemosum* mitogenome (**Supplementary Table S7**), spanning 23,278 bp with ≥80% sequence similarity to the plastome. These MTPTs account for 14.63% of the plastome and 2.57% of the mitogenome, respectively (**Figure 3**). Most MTPTs ranged from 30 to 500 bp in length, while five exceeded 1,000 bp, including the longest fragment (7,710 bp). Only six plastid-derived genes were retained within these MTPTs: five partial genes (*accD*, *rbcL*, *ccmC*, *trnL-CAA*, *trnY-GUA*) and one complete gene (*trnC-GCA*).

**Figure 4:**
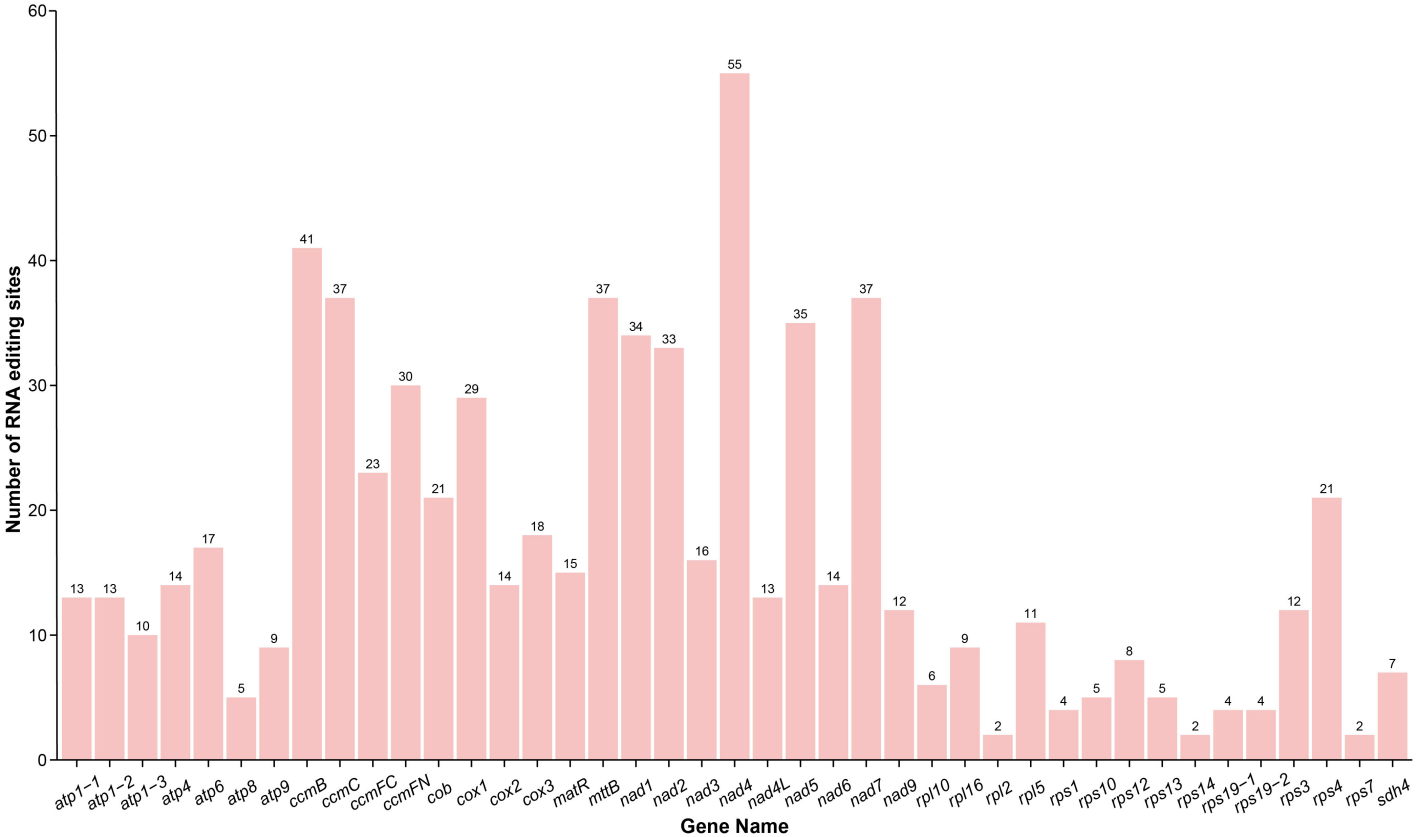
RNA editing events in the *Distylium racemosum* mitogenome. Characteristics of RNA editing sites across all protein-coding genes in the mitogenome of *Distylium racemosum*.

A total of 697 RNA editing events were identified in the PCGs of the *D. racemosum* mitogenome (**Supplementary Table S8**). The *nad4* gene displayed the highest number of RNA editing sites, with 55 events detected, whereas no RNA editing events were observed in the *sdh3* gene (**Figure 4**). RNA editing sites were distributed unevenly across codon positions: 229 (32.85%) occurred at first positions, 434 (62.27%) at second positions, and 34 (4.88%) at third positions.

### 3.4 Analysis of whole-genome collinearity

**Figure 5:**
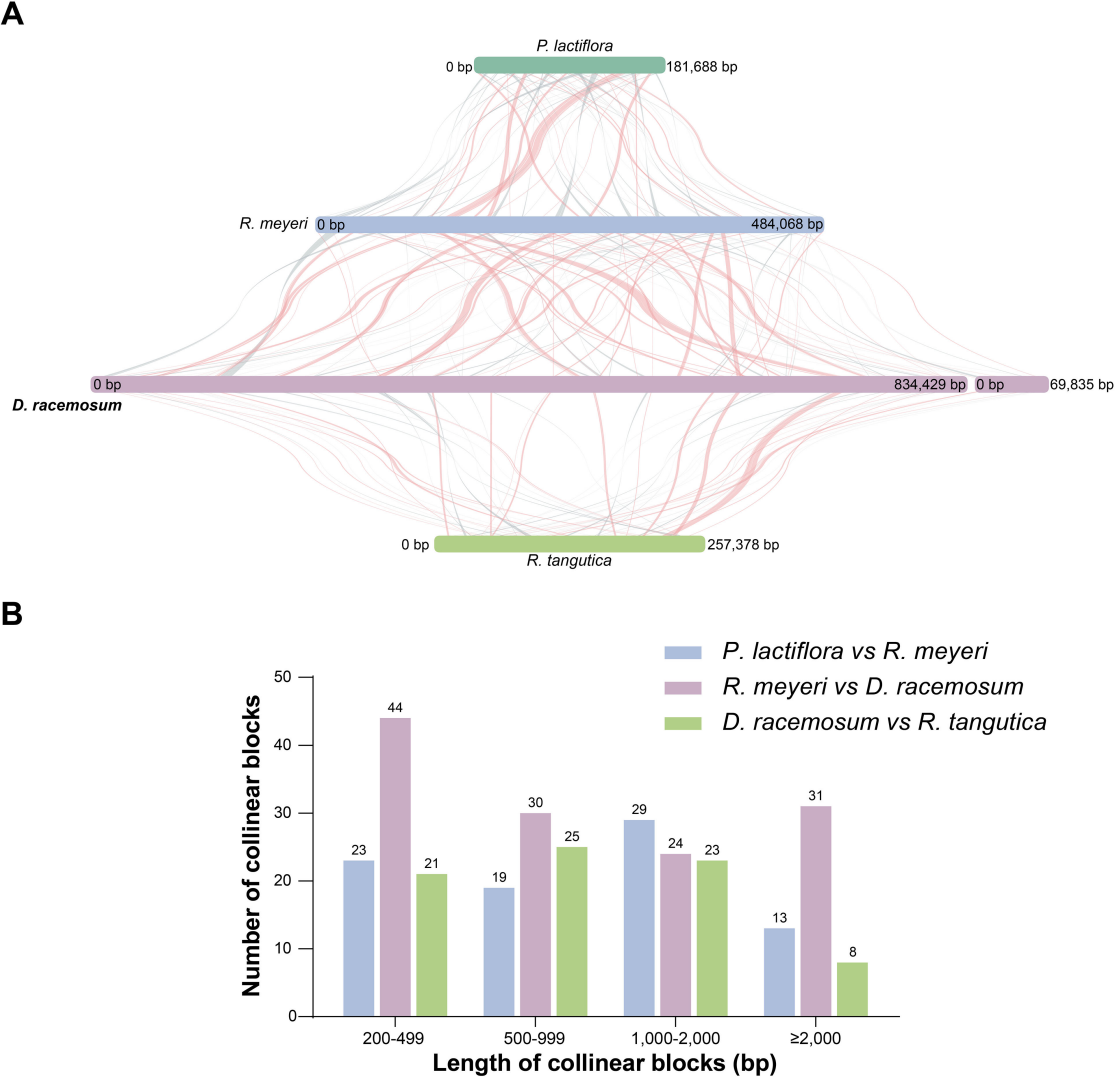
Whole mitogenome collinearity analysis of four Saxifragales species. **(A)** Collinearity analysis of the four Saxifragales mitogenomes. Strips of varying colors represented distinct mitogenomes, while linear blocks denoted collinear regions. **(B)** Distribution of collinear block lengths and counts. Color-coded legends indicated homologous fragments among the species. The names and NCBI accession numbers of species used in the collinearity analysis were provided in **Supplementary Table S1**.

We performed a comparative analysis to investigate the mitogenome collinearity among the four selected Saxifragales species. Extensive sequence rearrangements were evident between the *D. racemosum* mitogenome and those of the other species analyzed (**Figures 5A, B**). Between *D. racemosum* and *R. tangutica*, we identified 77 locally collinear blocks (LCBs; 81,121 bp), representing 31.52% of the *R. tangutica* mitogenome (**Figure 5B**; **Supplementary Table S9**). Similarly, a comparison of *D. racemosum* and *R. meyeri* revealed 129 LCBs, covering 183,570 bp and accounting for 37.92% of the *R. meyeri* mitogenome (**Supplementary Table S9**).

### 3.5 Phylogenetic analysis

**Figure 6:**
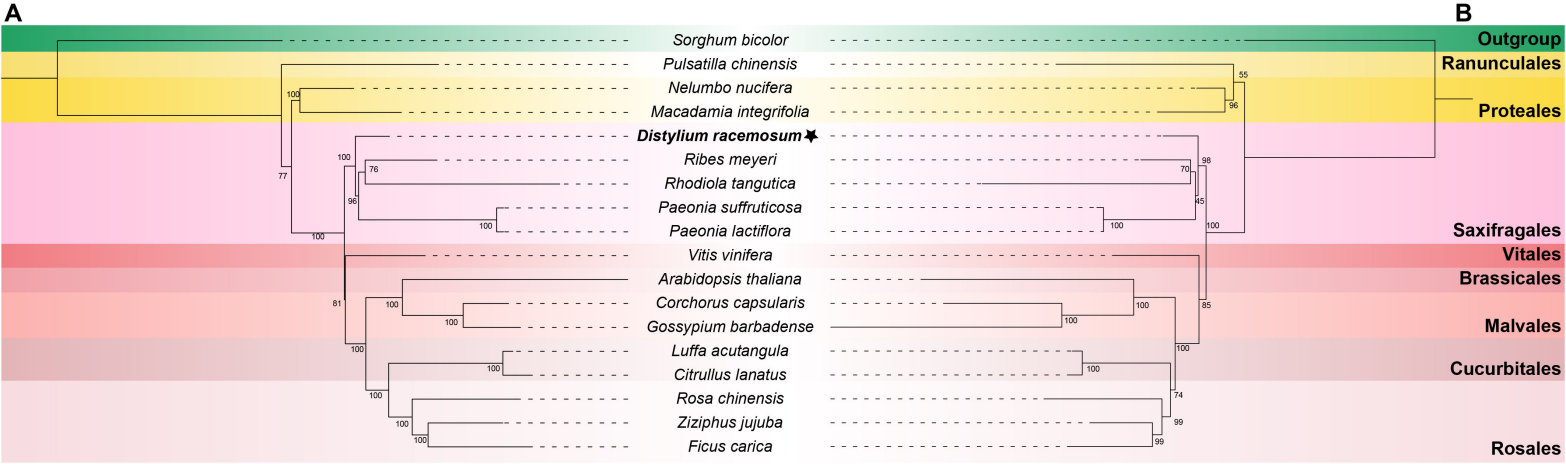
The ML trees of 18 plant species. **(A)** The ML tree based on 59 plastid PCGs. **(B)** The ML tree based on 18 mitochondrial PCGs. The mitogenome of *Distylium racemosum* was highlighted in bold and marked with an asterisk. *Sorghum* bicolor was chosen as the outgroup. Bootstrap values were indicated on each branch, and colors represented the respective groups for each species. The plant names and NCBI accession numbers utilized in the phylogenetic analysis were listed in **Supplementary Table S1**.

To determine the phylogenetic position of *D. racemosum*, a ML tree was constructed using 18 conserved mitochondrial PCGs from 17 plant species, with *S. bicolor* designated as the outgroup. The phylogenetic analysis revealed that *D. racemosum* clustered with four Saxifragales species (*R. meyeri*, *R. tangutica*, *P. suffruticosa*, and *P. lactiflora*), and occupied a basal position within the Saxifragales (**Figure 6A**). The topology received strong nodal support for the Saxifragales phylogeny (bootstrap value = 97). Notably, the five Saxifragales species were positioned at the base of the Superrosids, which is a sister group to the Rosids. To further validate our findings, we reconstructed the phylogenetic tree using plastome sequences. Phylogenetic reconstruction using plastid genomes (plastomes) yielded congruent results (**Figure 6B**), with topological congruence between mitogenome- and plastome-derived trees confirming the reliability of our phylogenetic inferences.

## 4 Discussion

### 4.1 Variety of structure and size in mitogenomes of Saxifragales species

Plant mitogenomes are organized into diverse structural conformations—including circular, linear, and complex branched molecules due to frequent recombination events (Bi et al., 2024b; Han et al., 2024; Wu et al., 2022). For example, *Amborella trichopoda*, *Rhopalocnemis phalloides*, and *Panax notoginseng* exhibit highly complex mitogenome architectures shaped by recombination (Rice et al., 2013; Yang et al., 2023b; Yu et al., 2022). Within Saxifragales, structural diversity of mitogenome is pronounced. *T. polyphylla* contains three circular chromosomes (Liu et al., 2024a), while species in *Rhodiola* genus display contrasting conformations—*R. crenulate* has a single circular chromosome, whereas *R. wallichiana* and *R. sacra* possess two circular chromosomes, reflecting atypical multi-chromosomal organization (Yu et al., 2023). Our study resolved the *D. racemosum* mitogenome as a bipartite structure comprising one large circular chromosome (834,429 bp) and a smaller linear fragment (69,835 bp). Recent research indicates that species with high GC content possess an enhanced ability to thrive in regions characterized by extremely cold winters or seasonal drought (Lu et al., 2024; Šmarda et al., 2014). The GC content in Saxifragales mitogenomes exhibits limited evolutionary variation, ranging from 44.50% (*Sedum plumbizincicola*) to 46.28% (*D. racemosum*) (Ding et al., 2022). The mitogenome size varies significantly across Saxifragales. In the Crassulaceae and Paeoniaceae families, the mitochondrial genome size ranges from approximately 180 bp to 260 kb, while Grossulariaceae and Saxifragaceae families span 400–500 kb, and *D. racemosum* (Hamamelidaceae) extends to 904,264 bp, which is the longest in Saxifragales to date, suggesting that Hamamelidaceae species may possess the larger mitogenomes among Saxifragales.

### 4.2 *D. racemosum* mitogenome exhibiting the highest repeats abundance in Saxifragales

Repeats are primary drivers of dynamic genomic restructuring in plant mitochondrial DNA, facilitating recombination-mediated changes in genome size, gene arrangement, and evolutionary trajectories (Wang et al., 2025; Wynn and Christensen, 2019). These structural modifications may ultimately contribute to phenotypic variation (Bi et al., 2022; Liu et al., 2024b; Tang et al., 2024; Wang Y. et al., 2024). For example, in *Zea mays*, repeats modulate chloroplast gene expression, directly affecting photosynthetic efficiency and growth (Bedbrook et al., 1977), while in *O. sativa*, repeat variations correlate with key agronomic traits like plant height and tillering capacity (McCarthy et al., 2002). In the *D. racemosum* mitogenome, we identified 304 SSRs, 1,508 dispersed repeats (748 direct; 761 palindromic), and 50 tandem repeats, representing the highest repeat density reported in Saxifragales mitogenomes (Liu et al., 2024a; Lu et al., 2024; Tang et al., 2024). This repeat abundance in *D. racemosum* provides a plausible explanation for its exceptionally large mitochondrial genome size in Saxifragales species (904,264 bp). These findings provide critical insights into *Distylium* evolution, underscoring repeats as drivers of genomic expansion and diversification.

### 4.3 Relatively abundant and stable RNA editing events in Saxifragales mitogenomes

RNA editing, a post-transcriptional modification critical for enhancing transcriptome diversity and producing functional mitochondrial proteins, is widespread across plant lineages (Edera et al., 2018; Gommans et al., 2009; Lukeš et al., 2021). Evolutionary trends reveal limited RNA editing in bryophytes (mosses and liverworts) but a marked increase in lycophytes (Rüdinger et al., 2009; Zhang et al., 2020). Among gymnosperms, the frequency of RNA editing demonstrates substantial variation, ranging from 99 editing sites in *Welwitschia mirabilis* to 1,405 in *Ginkgo biloba* (Fan et al., 2019; Guo et al., 2016). Angiosperms typically maintain moderate editing activity (400–500 sites). Notably, *Cinnamomum chekiangense* represents an exceptional case, exhibiting an unprecedented 1,119 RNA editing sites, the highest number recorded in angiosperms to date (Bi et al., 2016, 2024b). Within Saxifragales, studies conducted previously revealed substantial RNA editing activity: 569 sites in *P. lactiflora*, 653 in *Tiarella polyphylla*, and 731 in *R. nigrum* (Liu et al., 2024a; Lu et al., 2024; Tang et al., 2024). Our analysis identified 697 C-to-U editing sites across all PCGs except *sdh3*, underscoring both the prevalence and evolutionary conservation of RNA editing in this order. However, computational predictions necessitate experimental validation through PCR amplification and Sanger sequencing to confirm site-specific accuracy.

### 4.4 Low gene transfer level from plastome to mitogenome of *D. racemosum*

The transfer of plastid DNA sequences to mitogenomes, known as MTPTs, represents a recurrent evolutionary phenomenon in plant mitogenome evolution (Timmis et al., 2004). As a form of horizontal gene transfer (HGT), MTPTs exhibit considerable variation in length and sequence similarity across species (Tang et al., 2024). In this study, we identified 49 MTPTs in the *D. racemosum* mitogenome, with a cumulative length representing 2.57% of the total mitogenome. This proportion surpasses values reported for *P. lactiflora* (2.2%), *R. nigrum* (1.11%), *Arabidopsis thaliana* (0.8%), *Silene conica* (0.2%), and *Vigna angularis* (0.1%) (Lu et al., 2024; Sloan and Wu, 2014; Tang et al., 2024), yet remains lower than those of *Michelia figo* (5.53%) and *Boea hygrometrica* (10.5%) (Wang et al., 2025; Zhang et al., 2012). Gene retention within MTPTs also varies significantly. Only three plastid-derived PCGs were retained in *D. racemosum*—fewer than in *P. lactiflora* (10 PCGs), *R. nigrum* (12 PCGs), and *R. wallichiana* (7 PCGs), but more than in *R. crenulata* (2 PCGs) (Lu et al., 2024; Tang et al., 2024; Yu et al., 2023). The functional relevance of MTPTs appears minimal due to sequence degradation and lack of RNA editing (Clifton et al., 2004; Notsu et al., 2002), suggesting these genes likely have limited functional roles and represent nonessential genomic remnants (Wang et al., 2007).

### 4.5 Phylogenetic analysis of *D. racemosum* and its relatives

Plant mitogenomes demonstrate a remarkable propensity for the incorporation of exogenous or migratory DNA sequences, driving recurrent gains and losses of PCGs (Adams and Palmer, 2003; Garcia et al., 2021; Lu et al., 2024). In this study, we reconstructed two phylogenetic trees using 18 conserved mitochondrial and 59 plastid PCGs from 18 plant species. Both topologies strongly supported the basal divergence of *D. racemosum* within Saxifragales. Phylogenetic analyses further resolved Saxifragales as a basal lineage of Superrosids, emerging as a sister group to the Rosids, with tree architectures consistent with the Angiosperm Phylogeny Group IV (APG IV) system (Bremer et al., 2016). Nonetheless, limited mitogenome data from Hamamelidaceae species restricts deeper resolution of phylogenetic relationships within this clade.

## 5 Conclusion

In this study, we *de novo* assembled the first mitogenome of *D. racemosum*, comprising a 904,264 bp bipartite structure with a dominant circular chromosome (834,429 bp) and a smaller linear fragment (69,835 bp). The mitogenome encodes 67 genes, including 43PCGs, 3 rRNAs, and 21 tRNAs. Repetitive elements dominate the mitogenome, with 304 SSRs, 1,508 dispersed repeats, and 50 tandem repeats. Our analysis revealed 49 fragments (23,278 bp) that had been transferred from the plastome to the mitogenome of *D. racemosum*, representing 14.63% of the plastome and 2.57% of the mitogenome. We predicted 697 RNA editing sites across PCGs, predominantly at second codon positions (62.3%). Collinearity analysis revealed pronounced structural divergence between *D. racemosum* and other Saxifragales mitogenomes. ML trees reconstructed from mitochondrial and plastid genomes congruently resolve *D. racemosum* as a basal lineage within Saxifragales. As the first annotated mitogenome in Hamamelidaceae, this resource will advance comparative studies of mitochondrial evolution in Hamamelidaceae family and provide a genomic foundation for taxonomic refinement and applied research.

## Data Availability

The datasets presented in this study can be found in online repositories. These data can be found in Genbank repository, accession numbers for the mitogenome of D. racemosum are PQ594873 and PQ594874, respectively.

## Supplementary material

Supplementary Table 1 Names and GenBank accession numbers of species used for phylogenetic analysis.

Supplementary Table 2 Statistical Information of the *Distylium racemosum* mitogenome.

Supplementary Table 3 Start and stop codons of the *Distylium racemosum* mitogenome.

Supplementary Table 4 Distribution of simple sequence repeats (SSRs) in the *Distylium racemosum* mitogenome.

Supplementary Table 5 Distribution of dispersed repeats in the *Distylium racemosum* mitogenome.

Supplementary Table 6 Tandem repeats identified in the *Distylium racemosum* mitogenome.

Supplementary Table 7 Fragments transferred from plastome to mitogenome in *Distylium racemosum.*

Supplementary Table 8 RNA editing events predicted in the *Distylium racemosum* mitogenome.

Supplementary Table 9 Synteny results in mitogenomes of four Saxifragales species.

Supplementary Figure 1 The *cis*-splicing and *trans*-splicing introns in the *Distylium racemosum* mitogenome.

Supplementary Figure 2 Distribution of simple sequence repeats in the two mitochondrial chromosomes of *Distylium racemosum*.
