# Correction: *De novo* assembly and comparative analysis of the first complete mitogenome in *Distylium* (*Distylium racemosum*)

**DOI:** 10.3389/fpls.2025.1632449

**Published:** 2025-06-06

**Authors:** Yaling Wang, Zhongxiao Zhang, Xinru Chen, Honghe Li, Chi Ma, Penghui Guo

**Affiliations:** School of Life Sciences and Engineering, Northwest Minzu University, Lanzhou, China

**Keywords:** *Distylium racemosum*, mitochondrial genome, phylogenetic analysis, RNA editing events, comparative analysis

In the published article, there was an error in the **Funding** statement. The original funding text: “The author(s) declare that financial support was received for the research and/or publication of this article. The work is supported by the Northwest Minzu University Talent Introduction Project (Z2101707), the Science and Technology Department of Gansu Province.” The correct **Funding** statement appears below.

“The author(s) declare that financial support was received for the research and/or publication of this article. The work is supported by the Northwest Minzu University Talent Introduction Project (Z2101707), the Science and Technology Department of Gansu Province (23CXNA0045), the 2023 Central University Project (31920230027), and the Lanzhou Science and Technology Bureau (2023-RC-47).”

The authors apologize for this error and state that this does not change the scientific conclusions of the article in any way. The original article has been updated.

