# Supplementary figures and images for: De novo assembly and comparative analysis of the first complete mitogenome in Distylium (Distylium racemosum)
Source: Front Plant Sci. 2025 May 15;16:1586341. doi: 10.3389/fpls.2025.1586341 (PMC12119616; doi:10.3389/fpls.2025.1586341)

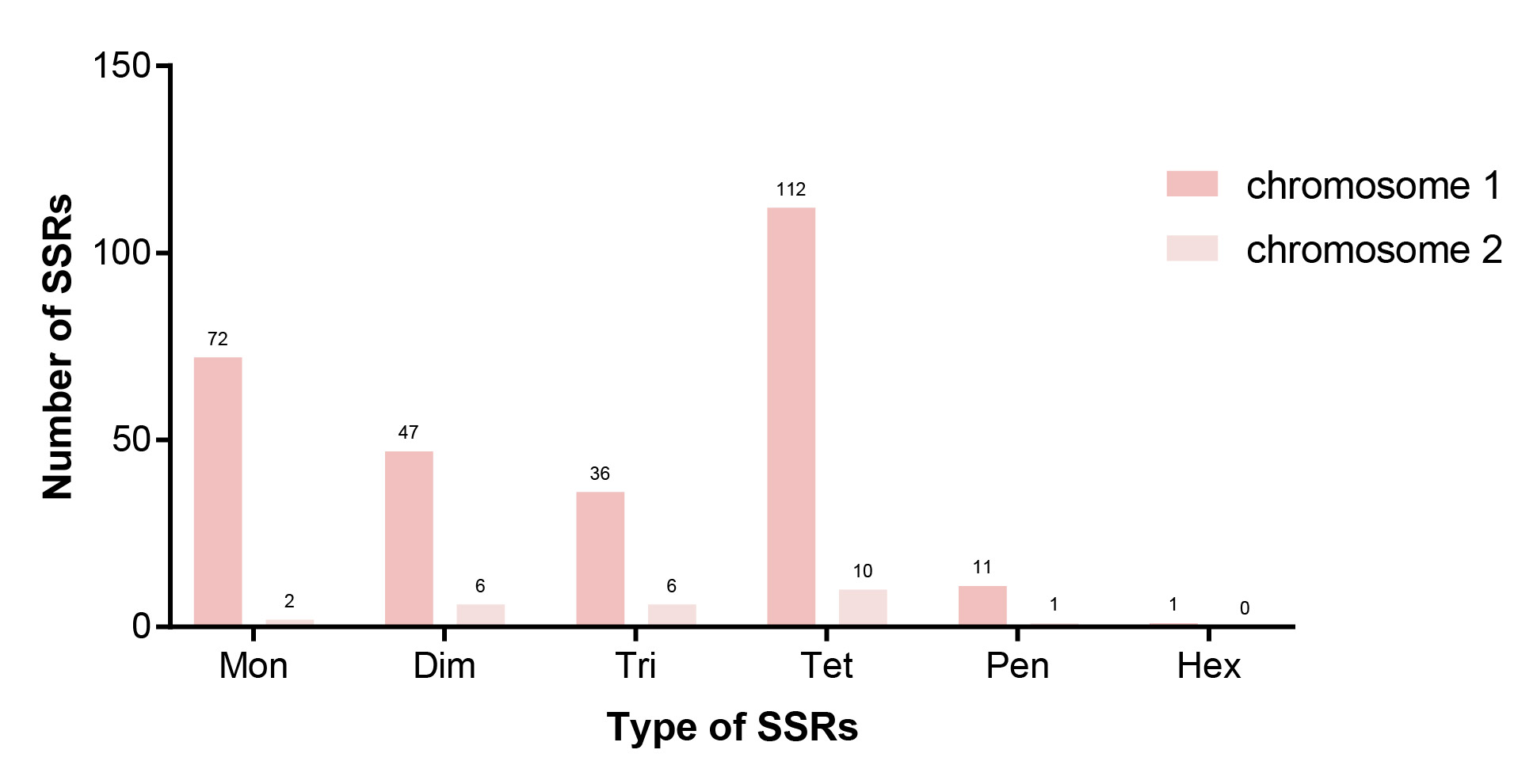

Supplement: Supplementary Figure 2 — Distribution of simple sequence repeats in the two mitochondrial chromosomes of Distylium racemosum. [file Image2.jpeg]
